# Applications of Electrospun Drug-Eluting Nanofibers in Wound Healing: Current and Future Perspectives

**DOI:** 10.3390/polym14142931

**Published:** 2022-07-20

**Authors:** Nakamwi Akombaetwa, Alick Bwanga, Pedzisai Anotida Makoni, Bwalya A. Witika

**Affiliations:** 1 Department of Pharmacy, Livingstone Central Hospital, P.O. Box 60091, Livingstone 10101, Zambia; anakamwi@yahoo.com; 2 Department of Surgery, University Teaching Adult Hospital, Private Bag RW 1 X Ridgeway, Lusaka 10101, Zambia; alick.bwanga@unza.zm; 3 Division of Pharmacology, Faculty of Pharmacy, Rhodes University, Makhanda 6140, South Africa; 4 Department of Pharmaceutical Sciences, School of Pharmacy, Sefako Makgatho Health Sciences University, Pretoria 0208, South Africa

**Keywords:** wound healing, wound dressing, sutures, drug-eluting nanofibers, electrospinning

## Abstract

Wounds are a consequence of disruption in the structure, integrity, or function of the skin or tissue. Once a wound is formed following mechanical or chemical damage, the process of wound healing is initiated, which involves a series of chemical signaling and cellular mechanisms that lead to regeneration and/or repair. Disruption in the healing process may result in complications; therefore, interventions to accelerate wound healing are essential. In addition to mechanical support provided by sutures and traditional wound dressings, therapeutic agents play a major role in accelerating wound healing. The medicines known to improve the rate and extent of wound healing include antibacterial, anti-inflammatory, and proliferation enhancing agents. Nonetheless, the development of these agents into eluting nanofibers presents the possibility of fabricating wound dressings and sutures that provide mechanical support with the added advantage of local delivery of therapeutic agents to the site of injury. Herein, the process of wound healing, complications of wound healing, and current practices in wound healing acceleration are highlighted. Furthermore, the potential role of drug-eluting nanofibers in wound management is discussed, and lastly, the economic implications of wounds as well as future perspectives in applying fiber electrospinning in the design of wound dressings and sutures are considered and reported.

## 1. Introduction

Wounds come about when the skin is damaged as a consequence of external mechanical forces, surgical operations, burns, chemical injuries, and ulcers from certain chronic diseases [1]. The skin functions as a waterproof insulating shield that protects the body against environmental stresses [2] and is divided into principle and distinct layers, viz., the epidermis, dermis, and hypodermis. These layers play various roles in thermoregulation, endocrine, metabolic, immunological, and neurosensory functions of the skin [3]. Once damage to the skin occurs, restoration of its integrity and function by way of wound healing is vital [4]. Replacement or repair of damaged or degenerated skin is required to restore the barrier function of the skin [5]. Wound healing is a complex process involving an assembly of mediators and various cells, leading to hemostasis, inflammation, proliferation, and remodeling [6]. The intensity of wound healing phases depends on the nature of the wound, where inflammatory and proliferative phases tend to be more critical in chronic wounds and surgical injuries than in minor wounds [7]. Wound healing stages occur in a systematic and overlapping fashion, in turn culminating in the repair of damaged tissue and restoration of integrity and function. Nevertheless, complications arise when the wound healing process does not conform to the predetermined order of events [8]. Factors such as systemic mediators, underlying disease, and the type of injury all affect the process of wound healing [6]. The prevalence of nonhealing chronic wounds in developed countries is estimated to be between 1 and 2% of the general population. Among these, surgical wounds and diabetic ulcers comprise the largest proportion [9]. Injuries account for the largest proportion of conditions where surgical treatment is indicated to either reduce preventable death or decrease the number of survivable injuries, resulting in personal dysfunction, which is likely to impose a significant burden on families and communities. Other conditions requiring primary surgical treatment are obstetric complications like obstructed labor and hemorrhage; a wide range of emergency abdominal and nonabdominal conditions, e.g., appendicitis; and several elective conditions that impact quality of life, such as cataracts [10]. Post-operative wound care is required for wounds to heal rapidly without complications and with the best functional and aesthetic results. Wound management typically involves keeping the wound clean coupled with use the of tissue approximating materials or wound dressings. The two common complications of surgical wounds are infection and dehiscence [11], with approximately 77% of surgical-related deaths being associated with surgical site infection (SSI) [12]. Owing to these statistics, infection prevention is pivotal in accelerating wound healing and preventing death. 

Healing of wounds with minimal tissue loss can be accelerated by approximating wound edges with sutures, staples, or wires, which results in healing by primary intention. Open wounds, on the other hand, present with excessive tissue loss and require a moist and sterile environment to heal optimally, which can be provided by wound dressings [8,13]. Modern wound dressings are designed to cover wounds and provide a barrier against bacterial penetration. Another type of modern dressings, called biomaterial scaffolds, obtained from natural tissues or artificial sources are biocompatible, biodegradable, non-toxic, and possess physicochemical properties that enable them to support cell attachment, proliferation and/or differentiation [14].

Scaffolding material originating from natural and synthetic polymers can be fabricated into film, foam, sponge, hydrogel, and nanofiber membranes. Scaffolds have found applicability in tissue engineering, where the materials are fabricated into artificial tissue that stimulates growth factors and enhances tissue regeneration. Among these materials, nanofibers possess a unique structure of small pore size and high porosity, thus protecting wounds from infections and ensuring unrestricted transportation of gas and liquid molecules [15,16]. Due to their unique properties, additional biomedical applications of nanofibers to wound dressing include drug delivery, vascular grafts, artificial organs, and tissue engineering [17]. Traditional fibrous wound dressings are fabricated by a spinning process where fibers or yarns are weaved, knitted, crocheted, or bonded in fabric. Modern wound dressings, however, are fabricated into non-woven textiles produced directly from the bonded fibers that are hot-pressed onto mats, producing more homogenous, softer, and more resilient structures with the added advantage of shorter processing time [18]. 

The electrospinning technique is recognized as a relatively simple and versatile method for producing functional nanofibers and nanofibrous membranes [19]. The use of the electrospinning technique to design nanofibers into wound dressing membranes provides versatility in terms of the characteristics of the wound dressings [18]. Fabrication of monofilament or multifilament sutures for tissue approximation using the electrospinning technique is also possible with the use of a rotating collector. When the electrospinning process is combined with braiding equipment, the produced nanofibers can be braided, making it possible to tailor mechanical properties and degradation rates [20].

Therapeutic agents are incorporated into nanofibers to produce bioactive wound dressings and sutures. Electrospun nanofibers are recognized as an advantageous material in drug delivery mainly due to their higher surface area with interconnected pores, thus providing better dissolution and high loading of therapeutics. Additionally, the rate of drug release can be tailored by tuning the fiber diameter, porosity, and drug-binding mechanism [19]. The administration of therapeutic agents accelerates wound healing and reduces the incidence of wound complications [20]. Therapeutic agents also augment the safety and efficacy of wound dressings and sutures [21]. Nonetheless, conventional administration of therapeutic agents is associated with some challenges, including the need for critical blood supply for adequate drug delivery in the case of systemic drug delivery and poor penetration of the stratum corneum, preservation of cell survival following drug delivery, and attaining effective, sustained drug delivery mechanisms in the case of local drug delivery [22]. These shortcomings can be overcome by the use of drug-eluting fibers [23]. Drug-eluting fibers provide localized drug delivery that potentially improves treatment through site-specific administration, ultimately reducing the dose of a drug required and incidences of systemic side effects [24]. The controlled elution of the drug can improve the safety of the fiber by modulating the local biological response [23].

A drug can be incorporated into a fiber either during production or onto an already fabricated fiber in post-processing. The former will involve melt extrusion or uni-/multi-axial electrospinning, where the drug and the carrier material are mixed together as a blend, suspension, or emulsion and extruded using heat application or electric charge. Post-process drug incorporation utilizes various techniques like dip coating, soaking, layer-by-layer deposition, supercritical carbon dioxide-assisted impregnation, embedding on a coated sheet, and grafting. The method of choice is influenced by the stability of the drug, desired drug loading, and physicochemical properties of the carrier material [20]. 

In this review, we discuss the process of wound healing, complications of wound healing, and current practices in accelerating wound healing. The potential role of drug-eluting nanofibers in wound management is discussed, and finally, future perspectives in applying fiber electrospinning techniques in the design of wound dressings and sutures are considered. 

## 2. Process of Wound Healing

Wound healing, a physiological process of the human body, comprises four highly integrated and overlapping phases. In summary, these phases include haemostasis, inflammation, proliferation, and remodelling [25]. Differences in time taken to achieve complete restoration are observed for different tissues [13]. For wounds to heal effectively, these precise and highly programmed phases must occur in a predetermined sequence and time frame at an optimum intensity [26].

### 2.1. Hemostasis

**Figure 1:**
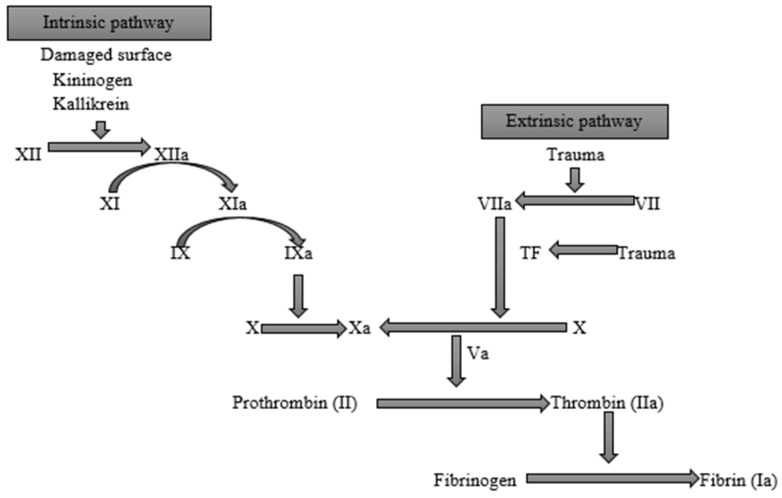
Coagulation cascade (Adapted from [31]).

When blood vessels are damaged, stopping blood loss is essential for survival. Hemostasis is achieved mainly through platelet adhesion and blood coagulation through a series of physiological cascades [27]. The initiation of coagulation is localized to cells that express tissue factors (TF) that are normally found outside the vasculature. Vascular injury exposes TF-expressing cells like adventitial fibroblasts to the blood and results in the complexation of TF with factor VII. The activated TF: factor VII complex, in turn, activates factors IX and X. This pathway leading to the activation of factor X is traditionally termed the extrinsic pathway because it occurs outside the endothelium [28,29]. The newly formed activated factor X (Xa) complexes with its cofactor (factor Va), resulting in the conversion of small amounts of prothrombin to thrombin. One of the main functions of the initially converted thrombin is platelet activation, which sets the stage for the intrinsic pathway. The intrinsic pathway is a parallel and complementary pathway that also leads to the activation of factor X. The activated platelets localize activated factors V, VIII, and IX onto their surfaces; the von Willebrand factor (vWf) then dissociates from factor VIII and mediates additional platelet adhesion and aggregation at the injury site [28]. When factor Xa is generated, it is incorporated into a prothrombinase composed of factors Xa and Va on the platelet surface, which results in a large burst of thrombin generation, which enhances the growth of the fibrin clot. The intrinsic and extrinsic pathways then converge into a common pathway that ultimately leads to the formation of fibrin subunits, which combine into fibrin strands that bind the platelets and stabilize the plug [30] as depicted in Figure 1. To localize clot formation to injured endothelium, a regulatory mechanism on endothelial cell surfaces through the protein C/protein S/thrombomodulin (TM) system acts by inactivating factors Va and VIIIa that may reach the intact endothelial cell [28]. The aggregated platelets in the fibrin plug then degranulate and release growth factors, including platelet-derived growth factor (PDGF), transforming growth factor-β(TGFβ), transforming growth factor-α(TGFα), basic fibroblast growth factor (bFGF), insulin-like growth factor-I(IGF-I), and vascular endothelial growth factor (VEGF). PDGF and TGFβ via chemotaxis then recruit neutrophils and monocytes from the vasculature to initiate the inflammatory response [26].

### 2.2. Inflammation 

The inflammatory phase is modulated by various chemical mediators, following clot formation and the control of blood loss. Endothelial cells are activated via cyclooxygenase 2 (COX-2) and synthesize prostaglandins that induce vasodilation, platelet disaggregation, and leukotriene synthesis [6]. The aggregated platelets degranulate and release potent chemo-attractants for neutrophils, macrophages, and lymphocytes that initiate the exudative and clean-up phase [25]. Typically, the first cells to migrate into the fibrin matrix are neutrophils, which are attracted by PDGF and other cytokines. Neutrophils phagocytose cellular debris, bacteria, and foreign material. Neutrophils primarily “mop up” the injury site by clearing debris and bacteria via the release of proteolytic enzymes and reactive free oxygen radicals [8]. The cells also release pro-inflammatory cytokines (including interleukin one and six (IL-1 and IL-6) and tumor necrosis factor-alpha (TNF-α) that recruit and activate fibroblasts and epithelial cells [26]. Neutrophils are, in turn, removed by physical sloughing or are phagocytosed themselves by macrophages [32]. Over the next two to three days, the population of inflammatory cells begins to shift to one of monocyte predominance. The monocytes are converted to macrophages that continue to phagocytose tissue and bacterial debris, as well as secrete multiple growth factors [8]. The conversion of monocytes to macrophages is important for the activation of the proliferative phase of healing [6]. The macrophages secrete collagenases that debride the wound. Other chemical mediators secreted by macrophages include PDGF, TGFβ, TGFα, fibroblasts growth factor (FGF), IGF-1, TNFα, IL-1, and IL-6. These growth factors and cytokines play a vital role in stimulating keratinocytes, recruiting, and activating fibroblasts and promoting angiogenesis [33]. The role of lymphocytes in the inflammatory phase is not well understood; however, studies suggest that delayed infiltration and low concentration of T-cells at wound sites are associated with impaired wound healing [25]. 

The mechanism of migration of inflammatory cells to the wound site is also vital in successful wound healing. The cells release elastase and collagenase to help them migrate through the endothelial cell basement membrane, with the fibrin clot serving as scaffolding for the arriving cells, which then migrate into the wounded tissue using their integrin to recognize and bind to extracellular matrix components [26,33]. Deficiencies in any one of these wound components impair the progression of the inflammatory phase to proliferative, ultimately delaying or preventing healing.

### 2.3. Proliferation

As the level of pro-inflammatory cells reduces following clean-up of debris, different cells are recruited to fabricate new tissue. The highlights of this phase are the restoration of structural function of the tissue, angiogenesis and formulation of granulation tissue, and epithelization [26]. Fibroblasts are the main cells and are recruited in the dermis for neovascularization and collagen deposition; these cells reform the clot into granulation tissue which provides physical and nutritional support for the repair of the upper layers. Keratinocytes also migrate and proliferate at the edge of the wound and extend the newly formed epithelial carpet that makes up the several layers of the epidermis [34]. The direction of movement of fibroblasts is determined by the concentration gradient of chemotactic growth factor cytokines and chemokines. PDGF and TGF-β are the two most important growth factors that regulate fibroblast activity. The former stimulates their proliferation, chemotaxis, and expression of collagenase, while the latter stimulates gene transcription of collagen, proteoglycan, and fibronectin as well as stimulates the synthesis of tissue inhibitors of metalloproteinases (TIMPs). Fibroblasts move along the alignment of the fibrils in the extracellular matrix and provisional matrix in the clot by changing their morphology which involves extending cytoplasmic projections to new binding sites, releasing the original site by local protease activity facilitated by released matrix metalloproteases (MMPs) and pulling themselves forward using their cytoskeleton network. Fibroblasts then synthesize and deposit collagen, proteoglycans, and other components of granulation tissue [26]. 

Angiogenesis is defined as the formation of new capillaries from existing small blood vessels; it is stimulated by various growth factors and cytokines, particularly vascular endothelial growth factor (VEGF) [26]. Angiogenesis normally accompanies cell proliferation, and new tissues cannot grow in its absence. The events leading up to the formation of new capillaries include local degrading of the basement membrane by proteases, migrating out of endothelial cells into a sprout, promotion of proliferation of endothelial cells that follow the sprouting cells by VEGF, and laying down of matrix around the new capillary [27]. The laid-down ECM provides scaffolding for cell migration, thereby contributing to the formation of granulation tissue [6]. The components of the ECM deposited by fibroblasts include collagen, fibronectin, hyaluronic acid, and proteoglycan. In-grown capillaries, lymphatic vessels, and components of ECM comprise the granulation tissue that provides skin strength and form [15].

Re-epithelization is the body’s means of re-establishing a protective barrier against fluid loss and bacterial invasion and is mediated by inflammatory cytokines [6]. During epithelization, fibroblasts are activated by myofibroblasts by macrophages, and myofibroblasts mediate contraction of the wound via actin and myosin action. Wound contraction is a consequence of cell bodies pulling together to decrease the area of tissue that needs to heal, which leads to the shortening of the scar [34]. The growth factors EGF, KGF, and TGFα bind to receptors on basal epithelial cells and stimulate their migration and proliferation. The desmosomes and hemidesmosomes that form intercellular connections between the basal epithelial cells with neighboring cells and the basement membrane, respectively, are dissolved once the growth factors bind to the cells so that the cells detach and prepare for migration. The normally cuboidal basal epithelial cells then flatten in shape and begin to migrate as a monolayer over the newly deposited granulation tissue along collagen fibers. The leading epithelial cells of the monolayer at the wound edges produce and secrete proteolytic MMPs that enable the cells to penetrate scabs, surface necrosis, or eschar. Once the epithelial cells from either end of the migration front converge, migration halts, and the proliferative mode begins, resulting in the re-establishment of the stratified layers, maturation, and restoration of the barrier function. Keratinocytes migrate and proliferate at the edge of the wound, which results in the extension of the newly formed epithelial layer comprising several layers of epidermal cells. Epithelialization is the clinical hallmark of healing; however, it is not the final event [26,34]. 

### 2.4. Remodeling 

The remodeling phase is characterized by processes that attempt to recover the normal tissue where the granulation tissue is gradually remodeled into scar tissue that exhibits an increased concentration of collagen fibers and is less cellular and vascular [35]. The initially laid down collagen is oriented parallel to the skin and is thinner than the basket weave patterned collagen found in uninjured skin. The granulation tissue collagen has greater hydroxylation and glycosylation of lysine residues that result in thinner collagen. To increase the tensile strength of the repair tissue, collagen type III is replaced by collagen type I, which is thicker and organized along the relaxed skin tension lines [6,33]. Cells in the wound bed produce proteolytic enzymes, specifically the MMPs: collagenases that degrade intact fibrillar collagen; gelatinases that degrade damaged fibrillar collagen molecules; and stromelysins, which very effectively degrade proteoglycans. Additionally, the serine protease neutrophilelastase degrades almost all types of protein molecules. The activity of these proteolytic enzymes is tightly regulated by specific enzyme inhibitors that are produced by cells in the wound bed. The MMPs are inhibited by TIMPs, while serine protease is inhibited by α1- protease inhibitor and α2 macroglobulin. Fibroblasts continue synthesizing collagen fibrils, which associate into larger bundles with time by forming stable covalent crosslinks that add to collagen strength [26]. Net collagen synthesis continues for approximately a month, despite the degradation and synthesis of well-organized collagen alongside the deposition of other proteins. Nonetheless, wounds never achieve the same level of tensile strength, with approximately only 80% of the initial tensile strength being achieved in the long term [6].

**Figure 2:**
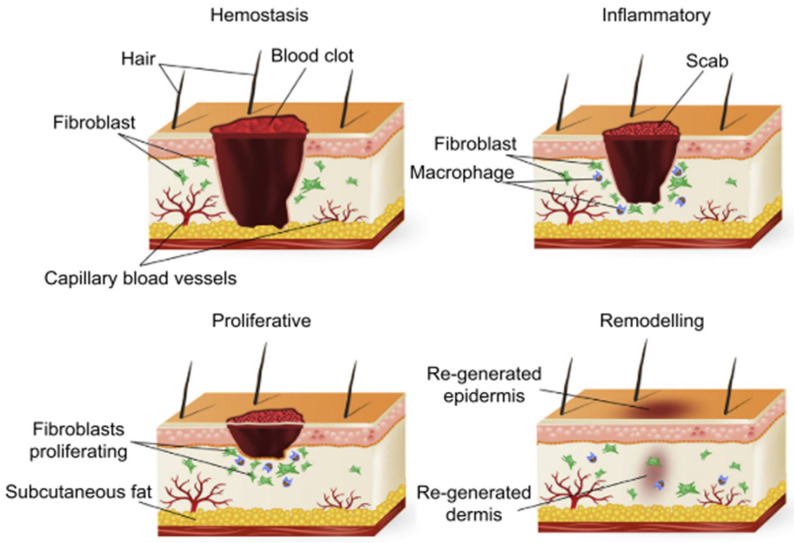
Stages of wound healing (reprinted from The Textile Institute Book Series, Erdem Ramazan, Advances in Fabric Structures for Wound Care—18, 509–540, 2019, with permission from Elsevier License Number-5271360368578).

Figure 2 depicts a summarized schematic of the previously described wound healing process [18].

**Figure 3:**
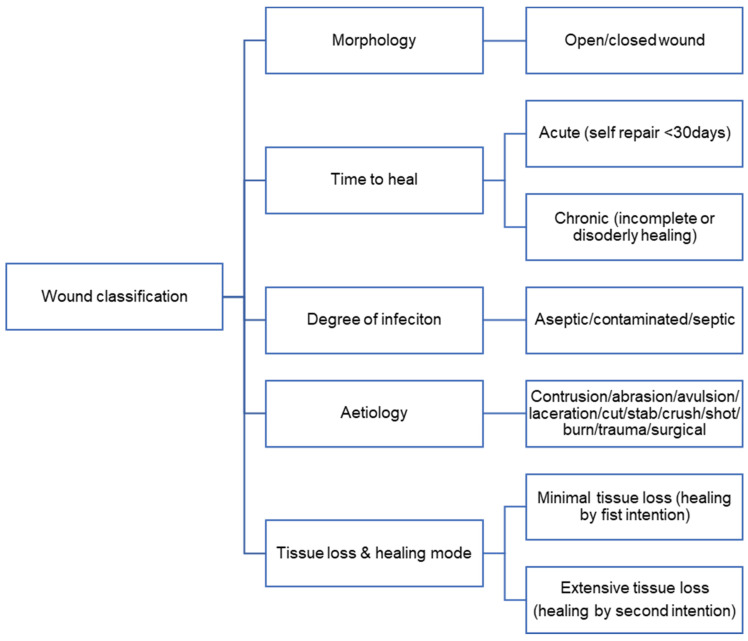
Wound classification criteria.

The rate and extent of wound healing depend on the characteristic of the wound. Classification of wounds is complex because various wound etiologies tend to present with inexact and overlapping features [36]. Wound types can be categorized using several criteria [13], as summarized in Figure 3.

## 3. Complications of Wound Healing

The phases of wound healing are distinct and occur simultaneously rather than sequentially, and failure in wound healing can occur because of a breakdown in any of these phases [37]. Consequences of wound healing failure include overactive wound healing or non-healing wounds, which are characterized by a stage of pathologic inflammation due to postponement, incomplete, or an uncoordinated healing process [37]. 

In the haemostasis phase, delayed or defective clotting mechanisms may lead to excessive blood loss, while an overwhelming inflammatory response can result in damage to non-injured tissue. The impaired activation of monocytes to macrophages in the inflammation phase results in defective wound repair; in the proliferative phase, disorders in wound contraction lead to deformity and the formation of contracture [6]. During remodelling, an imbalance in matrix degradation and synthesis can result in abnormal scar formation, such as hypertrophic or keloid scarring, which compromises the structure of the collagen matrix and adversely affects wound strength [6,18].

The process of tissue repair begins immediately after an injury and all wounds undergo similar phases of healing; however, specialized tissue like liver, skeletal muscle, and eyes have distinctive forms of regeneration. Different tissues also present with differences in the time required to complete regeneration [13]. Chronic wounds present as wounds that are non-healing or wounds that heal in a disorderly fashion. These wounds are usually related to neuropathy, vasculopathy, or trauma and can further be categorized as diabetic foot ulcers (DFU), pressure ulcers, venous stasis, or arterial insufficiency ulcers [38].

Other wound types that are difficult to manage include burns and scalds, radiation dermatitis, and split-thickness skin grafting (SSG). DFU is caused by neuropathy and lower extremity vascular disease. Stagnation in one or more phases of stages of healing can be caused by multiple factors. A reduced supply of oxygen and blood due to microvascular disease delays healing and increases infection risk [39]. Pressure injuries are caused by stress and tissue tolerance and are characterized by the occurrence of local injury of the skin or subcutaneous soft tissue at a site of bone prominence or compression of a medical device [40]. Burns and scalds are consequences of tissue damage caused by heat classified as surface, partial, or full thickness. They are characterized by the production of exudate, which increases in the inflammatory phase, eventually leading to delayed wound healing [41]. Chronic venous leg ulcers are caused by the high pressure of the blood in the leg veins, characterized by alteration in local blood circulation and tissue insufficiency [42]. Radiation dermatitis comes about when local skin lesions are caused by radiation; this results in slow cell proliferation decreased cytokine activity and decreased collagen content, all of which affect the healing rate [43]. SSG is a commonly applied reconstructive technique in the repair of orthopedic wounds and burns. If overlooked, the repair and regeneration of the donor site may result in infections, pain, and leakage that complicate and retard the healing process, as well as cause hypertrophic scars and hypopigmentation or hyperpigmentation [44].

### 3.1. Factors Affecting Wound Healing

There are many factors that could hinder or delay the extent of wound healing. Among these are:

- Infection—Microbial colonization of the wound site is the most common barrier to wound healing. It is universally accepted that all wounds have some bacteria present on their surface; however, detrimental colonization of a host organism by foreign species defines wound infection [36]. Signs of wound infection generally present as a lack of friable granulation tissue, excessive exudate, and degraded wound beds, which are more or less a consequence of stimulation of inflammatory cells by invading bacteria [15].
- Necrosis—Necrosis is categorized by the presence of dead skin and foreign material at the wound site [15]. Necrotic cells are mortally harmed cells whose loss of function and cellular integrity could potentially evoke irritation in surrounding tissue, ultimately leading to a persistent inflammatory state and stalled wound healing [45].
- Nutrition—Wound healing involves several biological and molecular events that can be impaired by poor diet. Macronutrients like proteins, carbohydrates, and fats are necessary for the repair of lost tissue [46]. Inadequate dietary protein results in decreased wound tensile strength and a diminished ability of the body to defend the wound against infection. Furthermore, micronutrients such as vitamins and trace elements are essential components of cellular function and deficiencies in these molecules can impair the modulation of the healing phases [47].
- Medicines—Molecules affecting any of the chemical signalling or cellular mechanisms of the healing process may impair healing [15]. Corticosteroids have been implicated in slowing wound healing by delaying the appearance of inflammatory cells, as well as cell growth and production [33].
- Moisture—Lack of moisture reduces tissue perfusion and slows down healing [15]. A moist environment is essential for the function of cells. Growth factors and other signalling molecules secreted following injury require a liquid medium for efficient intercellular communication. Epithelial cells also migrate and re-epithelialize more efficiently in a moist environment than in a dry one, saving tissue, time, and energy and reducing eschar formation [48].
- Individual physiologic variation–Patient conditions such as age and co-morbidities that restrict blood flow and hinder the activity of the immune system can cause non-healing of wounds [15]. Diabetes, obesity, hypothyroidism, and stress are associated with the development of chronic wounds. Conditions that present with impaired renal, hepatic, or respiratory function also hinder the wound healing process [33].

### 3.2. Wound and Scar Formation

Scar formation is a consequence of wound healing. The spectrum of wound closure ranges from scarless regeneration on the one end to normal scar formation and pathological scar formation on the other end [32]. Scarless regeneration is observed in smooth, closed abutting wounds where tissue loss is minimal. Deeper wounds with significant tissue loss are closed by the growth of new granulation tissue, which eventually transforms into scar tissue [49]. The lost tissue is partially regenerated by hyper-production of collagen in a series of deposition and absorption of the fibrous matrix that results in, at most, 80% similarity with the original tissue [33]. Accelerating the rate of wound healing minimizes scar formation and requires the promotion of wound closure without tension, prevention of infection, and wound breakdown. In general, there is a quality-of-life burden on individuals associated with scarred skin as a result of burns and surgery [32]. It is estimated that 100 million patients globally acquire scars each year, and over half of these result from elective and post-trauma operations [50]. Abnormal skin scarring has physical, aesthetic, psychological, and social consequences, which may result in substantial emotional and financial costs. It is estimated that the annual market for scar treatment amounts to approximately USD 12 billion in the United States alone [32]. In particular, children can suffer from long-term physical dysfunction and psychological harm from scars that result from major burns and surgery. Patients with visible scars, especially on the face, tend to suffer from social stigma and psychological trauma. Furthermore, a large proportion of patients treated for burns in the United States end up with scars and painful contractures that require major surgery [32]. 

Scarless tissue repair has been observed in a mammalian foetus and is thought to be a consequence of differences between the extracellular matrix (ECM), inflammatory response, cellular mediators, differential gene expression, and stem cell function in foetal vs. adult wounds [51]. The ability to heal scarless is lost with advancing gestation age, and ECM composition is considered to be a contributing factor. Foetal skin has a higher ratio of type III to type I collagen than adult skin and contains more hyaluronic acid. The proteoglycan ECM modulators, decorin, lysyl oxidase, and MMP are present in considerably larger amounts in mature skin, and fibromodulin production decreases with maturation [52]. Research advances show that rapid epithelization and alteration of levels of growth substances and their inhibitors could possibly enable manipulation of the wound healing process in adults to produce more foetal-like tissue repair [33]. 

## 4. Accelerating Healing of Surgical Wounds

Surgery can be defined as the branch of medicine that employs operations in the treatment of disease or injury. Surgery involves cutting, abrading, suturing, or otherwise physically changing body tissues and organs [53]. It is estimated that 11% of the daily adjusted life years (DALYs) associated with the global population are from conditions requiring surgery [10]. In addition to its application in treating injuries, surgery is a key element in primary health care. Surgery is an essential intervention in limiting maternal and child mortality and is also applied in draining abscesses and management of traumatic joint dislocation [54]. Traumatic injuries account for the largest proportion (38%) of the burden of common surgical conditions [10]. Following surgery, post-operative wound care is essential to achieve rapid wound healing without complications and with the best functional and aesthetic results. The use of dressings, wound cleaning, antibiotic treatment, and debridement are recommended practices that aid in the reduction of SSI [11]. To perceptively identify patients at risk of SSI, the Centers for Disease Control and Prevention (CDC) recommend the utilization of a surgical wound classification (SWC) system [55]. Wound types are categorized as follows:

“*Class I/Clean: An uninfected operative wound in which no inflammation is encountered and the respiratory, alimentary, genital, or uninfected urinary tract is not entered. In addition, clean wounds are primarily closed and, if necessary, drained with closed drainage. Operative incisional wounds that follow nonpenetrating (blunt) trauma should be included in this category if they meet the criteria*.

*Class Il/Clean-Contaminated: An operative wound in which the respiratory, alimentary, genital, or urinary tracts are entered under controlled conditions and without unusual contamination. Specifically, operations involving the biliary tract, appendix, vagina, and oropharynx are included in this category, provided no evidence of infection or major break in technique is encountered*.

*Class Ill/Contaminated: Open, fresh, accidental wounds. In addition, operations with major breaks in sterile technique (e.g., open cardiac massage) or gross spillage from the gastrointestinal tract and incisions in which acute, nonpurulent inflammation is encountered are included in this category*.

*Class IV/Dirty-Infected: Old traumatic wounds with retained devitalized tissue and those that involve existing clinical infection or perforated viscera. This definition suggests that the organisms causing postoperative infection were present in the operative field before the operation.*” [56].

Classification of wound type does not always accurately predict nor prevent SSI, as observed in a study that assessed the association between the SWC and the rate of subsequent SSI in orthopedic trauma cases. The study reports that patients with diabetes and those with lower extremity injuries were significantly at higher risk of developing SSI. However, host-dependent variables like postoperative patient compliance, wound surveillance, and acute rehab versus home care were not controlled for. It was suggested that the lack of direct or indirect association between the SSI rate/incidence and SWC grade might be due to current operating room standards, surgical techniques, perioperative wound protocols, improved efficacy of antibiotics, and active surveillance of high-risk patients like those with open injuries [57]. One of the major problems with SWC is its failure to account for the intrinsic patient risk of developing an SSI. Literature shows that the national nosocomial infections surveillance (NNIS) basic SSI risk index is a significantly better predictor of SSI risk than the traditional WCS. The NNIS basic SSI index combines the following three variables: level of contamination of the surgical wound, the American Society of Anesthesiologists (ASA) pre-anesthesia score, and the duration of the surgical procedure. A number of studies have reported a decrease in the incidence of SSIs when surveillance programs have been implemented in adjusting for risk for most procedures [58]. Reducing infection of surgical sites is reported to have benefits such as reduction in postoperative morbidity and mortality and the wasting of healthcare resources [59]. Table 1 shows a summary of results obtained by hospitals in various countries implementing surveillance systems that provide insight into the incidence of SSI. 

Good predictors of SSI development include type, number, and length of surgery; the patient’s medical history and severity of the current condition; whether the surgery is elective or emergency; and whether anesthesia is general or local [63]. Table 1 suggests that superficial infections occur more frequently than deep incisional and organ-specific infections, highlighting the relevance of the barrier function of the skin. Surgical procedures indicated in these studies range from abdominal surgery in adults and neonates [61,64,66] to caesarean section [65] and orthopaedic procedures [57]. The presence of comorbidities like diabetes, hypertension, and obesity are associated with higher SSI incidence, while the administration of antibiotics pre-operatively and the use of laparoscopy shortening the duration of the procedure present a reduction in SSI incidence [57,58,60,65]. Ultimately immunological function, blood perfusion, and nutritional status play important roles in infection prevention, particularly when exposure to microbial infiltration is prolonged. Functional wound dressings and sutures that accelerate restoration of barrier function become important components of wound care management. Dressings suitable for different wound types are categorized as protective, which include gauze and impregnated gauze; antimicrobial dressings such as ointments and iodine or silver-based dressings; absorbent dressings, which include foam, hydrogel, and hydrofibers; and autolytic debridement dressings, which comprise films, hydrogels and hydrocolloids [11]. Wound dressings are applied to wound surfaces to promote wound healing [67]. Modern wound dressings utilize biomaterials whose applications include the provision of a native environment and supportive matrices that induce the growth of tissues; the creation of physical obstacles against microbial contamination; and delivery of therapeutic agents. Approaches to induce and accelerate wound healing can be summed up in terms of cell therapy, bioactive therapeutic drug delivery, and the use of biomaterials. A combination of these strategies potentially provides an ideal approach to wound management. In cell therapy, new skin is generated using multipotent stem cells and co-culture techniques; engineered bioactive therapeutic delivery systems release healing components that enhance the rate of wound repair, and scaffolds are applied in tissue engineering and induction of regeneration [68]. These dominant strategies for accelerating and inducing wound healing are discussed in detail, and current practices and challenges associated with their use are also addressed.

### 4.1. Cell Therapy

Seven stem cell populations have been considered so far for their potential use in wound healing and include mesenchymal stem cells (MSC) derived from skin, MSC from bone marrow, MSC from adipose tissue, stem cells derived from cord blood, stem cells derived from extra foetal tissue, embryonic stem cells, and induced pluripotent stem cells [69]. MSC have the ability to differentiate into multiple tissue-forming cells. They also exhibit immunomodulatory, reparative, and regenerative effects through paracrine signalling [70]. The multipotent nature of stem cells results in accelerating wound closure, enhancing re-epithelialization, increasing angiogenesis, promoting granulation tissue formation, modulating inflammation, and regulating ECM remodelling, thereby demonstrating a beneficial effect on cutaneous wound healing and skin regeneration. Stem cells characteristically are capable of self-renewal, asymmetric replication, and differentiation into other cells, thus building different tissues and organs. They replenish the lost cells of an organism, thereby maintaining the number of aging somatic cells [71]. The therapeutic potential of stem cells largely depends on their capability to secrete pro-regenerative cytokines. Stem cells are, however, associated with potential immunogenicity and tumorigenicity, as well as poor engraftment efficiency and cell retention at the wound site, which limits their therapeutic potential [70]. Other challenges to therapeutic potential are related to determining the optimum source and method of processing, as well as administration from a clinical standpoint [69].

### 4.2. Bioactive Therapeutic Delivery

Pharmacologically active agents of various classes such as growth factors, gene-therapy [33], antimicrobials, anti-inflammatories [18], bio-adhesives, plant extracts, and animal products are used as adjuvants of wound healing [72]. However, due to limitations associated with systemic drug delivery like low bioavailability, toxicity, and significant side effects caused by non-target delivery, the use of localized target delivery provides a better prediction of drug release patterns for a given therapeutic window [68]. 

Growth factors considered necessary for wound healing include epidermal growth factor (EGF), fibroblast growth factor (FGF), insulin-like growth factor (IGF), keratinocyte growth factor (KGF), platelet-derived growth factor (PDGF), transforming growth factor (TGF), and vascular endothelial growth factor (VEGF) [18]. Among these, only PDGF-BB has been approved for clinical use and is available as becaplermin (0.01% Regranex^®^ gel, Smith & Nephew, Fort Worth, Texas, USA). The limitation in the development of growth factors is attributed to limited data in humans as a consequence of the difficulty in controlling or conducting clinical studies [73]. An alternative to growth peptide administration is inserting growth factor genomes into the wound. Various techniques of gene therapy are applied for this purpose, most of which have limitations of low levels of transfection or adverse immune response despite showing accelerated wound healing response [74].

Infection control should be carefully considered in the management of wounds and is recognised as a measure of the quality of care of patients [59]. The use of appropriate antimicrobial agents in addition to aseptic techniques and optimal debridement is required for infection control [75]. Antimicrobial agents consist of disinfectants, antiseptics, and antibiotics. Antiseptics and antibiotics are both used on intact skin and wounds; however, the use of antiseptics in open wounds is limited [38]. Antiseptics have a broader spectrum of activity against microorganisms than antibiotics but show higher cytotoxicity, while antibiotics have a higher probability of bacterial resistance development. Local adverse effects associated with antibiotics include pain, rash, and cytotoxic effects on the cells such as fibroblasts, epithelial, endothelial, and inflammatory cells [75].

Inflammation is one of the distinct phases of wound healing, with the chemical mediators released during this phase, setting the stage for initiation of the proliferative phase [8]. Anti-inflammatory steroids have a direct inhibitory effect on the fibroblast genome and generally inhibit cell growth and production, resulting in widespread negative effects on wound healing [33]. A stage of pathologic inflammation on the wound bed results in a chronic or non-healing wound [37]. Therefore, the use of anti-inflammatory agents may be required to limit the emergence of wound complications. Non-steroidal anti-inflammatory agents (NSAIDs) are routinely used as post-surgical treatment due to their antiphlogistic, antipyretic, and analgesic effects [76]. The impact of NSAIDs on wound healing is highly controversial, considering that they inhibit cyclooxygenase, an enzyme that catalyses the synthesis of prostaglandin E_2_, which mediates inflammation and modulates fibroblasts [77]. Ascertaining the exact impact of NSAIDs on wound healing is, however, challenging due to the lack of an in vivo model for the study of specific phases of the wound healing process [77]. A study on the use of diclofenac in wound healing reports that the administration of the agent did not affect the macroscopic and microscopic wound healing outcome. Furthermore, despite the observed decrease in the population of fibroblasts in the connective tissue, NSAIDs can be recommended for post-surgery and post-traumatic wounds due to their antiphlogistic and analgesic effect [76]. 

Bioadhesives are described as natural or synthetic materials that adhere to biological elements such as cells, tissues, and organs through physical or chemical conjugation [78]. Clinical applications of bioadhesives include wound closure, sealing of blood or other fluid leaks, immobilization, which involves wound dressing, drug/cell delivery, or device fixation in tissues [79]. The mechanism of tissue adhesives is explained by molecular bonding, which is characterised by interatomic and/or intermolecular forces established between the molecules at the tissue surface and the molecules of the adhesive [80]. In wound closure, bioadhesives are considered a good non-invasive alternative to sutures, wires, and staples [79]. Natural bioadhesives are derived from natural polymers that comprise the protein or polysaccharide cross-linkages [80]. Protein-based bioadhesives include fibrin, collagen, and gelatine-based polymers, while polysaccharide-based bioadhesives include chitosan, alginate, and chondroitin-based polymers [81]. Another class of bioadhesives are nature-inspired bioadhesives that include mussel-mimetic polymers inspired by mussel adhesive proteins (MAP) and gecko-inspired nanoscale pillars. These nature-inspired bioadhesives are, however, yet to become commercially available [78]. Examples of synthetic and semisynthetic bioadhesive agents include cyanoacrylate-based adhesives, polymeric hydrogels, dendrimers, and urethane-based adhesives [81]. Natural bioadhesives are generally more biocompatible than synthetic glues but tend to exhibit reduced mechanical strength. Synthetic and semisynthetic bioadhesives have low bio-absorption and metabolism, low adherence to wet surfaces, and higher cytotoxicity [80].

Medicinal plants like *Cestrum nocturnum, Guiera senegalensis, Commelina diffusa,* and *Spathodea campanulata* are used traditionally to treat wounds. However, the crude plant extracts may contain phytochemicals that might be potentially harmful to exposed tissue and detrimental to the wound healing process [82]. One study fabricated a curcumin-loaded wound dressing that demonstrated acceptable safety and wound healing efficacy [83]. Another study showed that topical application of Cestrum nocturnum significantly (*p* < 0.05) promoted wound healing [84]. These studies involved superficial wounds; therefore, safety and efficacy data would have to be determined for deep incision wounds. 

Animal-derived products that have been considered adjuvants for wound healing include bovine-derived amniotic membrane and intestines, silk protein, and fish skin. The major limitations with natural products include batch-to-batch variability, long-term immunogenicity, and safety [72].

Cell biology is modulated by cell/tissue response to their microenvironment via a myriad of biochemical and biophysical cues. These include cell surface proteins like integrin and other receptors transducing chemical and mechanical cues into biochemical signals following direct interaction with microenvironment conditions of chemical composition, matrix stiffness, and topography. Extracellular growth factors, cytokines, chemokines, and natural products like collagen and fibronectin are capable of inducing cytoskeleton rearrangement affecting cell adhesion and spreading and enhancing cell adhesion and growth [85]. Gas concentrations (reactive oxygen species; ROS) in the microenvironment also mediate intracellular chemical signaling resulting in proliferation, migration, and differentiation indirectly. Very high concentrations of these gases, however, may result in pathological diseases due to the oxidation of intracellular components like proteins [86]. The responses of the cell/tissue microenvironment provide a myriad of biochemical and biophysical cues for modulating cell biology, which sheds light on tissue regeneration and disease therapies [87]. To mediate a radical attack of cells by oxidizing species, the use of metallic nanoparticles and nano-enzymes, such as gold nanoparticles and lead nanocrystals, have been recommended. The clinical application of artificial nanomaterials is, however, limited due to long-term instability and potential toxicity under physiological conditions. Consequently, natural or natural-inspired biomolecules are considered safe and efficient antioxidants in biological tissues [88]. These natural and synthetic polyphenolic macromolecules possess unique structural and functional properties. Polyphenolic materials comprise active functional groups like catechol and pyrogallol that allow tuning of physicochemical properties via chemical modification. Polyphenols also have ROS scavenging and photothermal properties, making them promising candidates for the regulation of extra- and intracellular ROS levels and temperatures and ultimately influencing cell biology. Polyphenols can consequently be applied to accelerate wound healing [87]. Melanin belongs to a class of natural biomolecular pigments utilized as a theranostic antioxidant and thermal stabilizers of polymers due to their excellent free radical scavenging capabilities. Melanin-inspired polydopamine (PDA) is prepared by oxidation polymerization of dopamine and possesses a similar structure to the predominant eumelanin. The melanin-mimicking PDA shows similar chemical, physical, and biological properties as natural melanins in antioxidation, photoprotection, metal chelation, and energy dissipation [89]. One study demonstrated that the incorporation of PDA nanoparticles in a dextran-based wound dressing promoted wound healing by inhibiting the growth of bacterial colonies and alleviating oxidative stress. Tissue regeneration was enhanced due to the timely and complete progression of all the phases of wound healing [90]. 

### 4.3. Scaffolds/Biomaterials

Traditionally, wound dressings like gauze bandages or highly absorbent cotton wool pads were used to cover wounds; however, they have been replaced by scaffolds because of inefficient mechanical properties and bacterial protection [15]. Scaffolds allow cell attachment and migration, modify the diffusion of vital cell nutrients, and exert certain mechanical and biological influences to modify the behaviour of the cell phase [15]. Most cells in human tissue are encourage-dependent and reside in the ECM [91]. Scaffolds are used in tissue recovery to provide the epidermis with a base for wound healing elasticity and proper strength that feed the keratinocytes in the epidermal layer. The construction of tissue scaffolds is among the crucial elements in skin tissue engineering [15]. Scaffolds are three-dimensional (3D) porous solid biomaterials designed to provide spatially correct cell location, promote cell-biomaterial interactions, cell adhesion and ECM deposition, and permit sufficient transport of gases, nutrients, and regulatory factors. Ideally, scaffolds should biodegrade at approximately the tissue regeneration rate and provoke minimum inflammation and toxicity in vivo [91]. 

Scaffolds are made of either natural or synthetic biomaterials present in the form of films, hydrogels, or nanofibers, among others [68]. Films are composed of transparent and adherent polyurethane that permits permeation of water and gases at the wound site and provides autolytic debridement of eschar and a microbial barrier [14]. Films tend to obstruct the regeneration of epithelium and present with low mechanical strength. In contrast, nanofibers are similar to natural ECM and provide an ideal microenvironment for cell adhesion, proliferation, migration, and differentiation. Consequently, nanofibers are gaining consideration as an advanced scaffolding material [1]. Hydrogels are water-absorbing hydrophilic and highly flexible polymeric materials [48]. Hydrogels rehydrate dry wounds and do not cause damage when removed; however, they require secondary dressing and tend to get saturated [49]. Fibers with diameters in the nanometric range exhibit large specific surface areas, high porosity, and small pore size [92]. 

The modern dressings that include hydrocolloids, hydrogels, alginates, polyurethane foam/films and silicone gels can be used to deliver active agents to wounds [82]. These materials comprise natural and synthetic polymers, which can be fabricated into polymeric drug carriers capable of providing therapeutic drug delivery in addition to stimulating biologic responses of tissues. Techniques used in fabricating polymeric drug carriers include polymerization, interfacial polymerization, copolymerization, polymerization mixing, dissolution precipitation, phase inversion, hemispheres, solvent evaporation, solvent casting, micro-molding, hot embossing, and magnetorheological drawing lithography (MRDL) [93]. 

Suspension, interfacial, bulk, and emulsion polymerization are mostly used in the preparation of microparticles. The bulk polymerization method involves heating one or more monomers in the presence of a catalyst; the process is simple and is usually achieved in a single step [94]. Interfacial polymerization occurs at the interface of two immiscible liquids. Films fabricated by this method under controlled conditions are inherently uniform and free of defects, with the process taking time, and the polymers are costly [95]. Copolymerization involves the synthesis of copolymers that enable improving properties of polymeric material for industrial application [96]. The mixing process is used in the preparation of raw polymers, which usually requires the addition of fillers, and production is costly [97]. 

The precipitation process allows for the formation of pure and homogenous material. In dissolution, the solute particles separate from each other and become surrounded by the molecules of the solvent. The limitation of this process is the need to separate the drug carrier and the use of bulky solutions [93]. Phase invasion is mostly used for making polymeric membranes. The process involves controlled polymer conversion from the liquid phase to the solid phase [98]. Solvent evaporation and solvent casting also involve the use of polymer while applying specific solvent removal techniques [93]. Hemispheres provide a means of developing reservoir-controlled drug release delivery methods; the production of the hemisphere involves coating core material with an insoluble polymer coating using conventional spray film coating techniques [99]. Micro-molding, hot embossing, and MRDL are techniques that are better suited for drug delivery to intact skin, considering that most are fabricated into microneedle arrays [93] and could therefore not be appropriate for application to open wounds.

#### Nanofibers

The rapid increase in research on nanofibers has led to the consideration of nanofibers as potential candidates for wound dressing application [49]. Nanofiber sutures have also gained research interest because of their inherent advantage of being able to prevent adherence of bacteria to the filaments as a consequence of their size [100], with wider diameter sutures having a higher risk of bacterial adherence [101]. Nanofiber sutures produce less tissue reactivity, mimic collagen fibers in the extracellular tissue matrix, and provide the cells with a native environment at the wound site with a reduced inflammatory response. Nanofibers provide a high surface-to-volume ratio for drug elution and interaction with cells that accelerate healing [19]. The use of nanofibers as delivery systems for bioactive agents at the wound site, in addition to the provision of mechanical support, provides two out of three domains of inducing and accelerating wound healing, potentially resulting in successful tissue repair or regeneration [12]. 

The most commonly used natural polymers in nanofibrous scaffolds are chitosan, collagen, gelatin, and silk, while synthetic polymers include polycaprolactone (PCL), poly-lactide acid (PLA), and poly(lactic-co-glycolic acid) (PLGA) [102]. Nanofibers are fabricated using either mechanical or electrostatic force. Techniques involving the use of mechanical force include phase separation, drawing, template synthesis, or self-assembly. The electrospinning technique uses electrostatic force and is the most commonly used technique because of its simplicity and versatility [103]. Electrospinning permits the fabrication of multicomponent nanofibers that are morphologically and mechanically more analogous to native ECM [19]. This is an important feature because natural polymers, despite being biocompatible and biodegradable, have unfavourable mechanical characteristics, limited applicability, and degrade fast, while synthetic polymers are reproducible and inexpensive but lack biomimetic features [80]. 

When used for drug delivery, properties of electrospun nanofibers can be tailored to control the rate of drug release by tuning fiber diameter, porosity, and drug-binding mechanism [19]. Nanofibers present an opportunity to modify the release of therapeutic agents, which may be desirable in regulation of inflammation and remodeling and infection prevention. Sustained drug release is influenced by the fiber diameter, porosity of the fiber, and drug-binding mechanism [19]. The drug-binding mechanism appears to consistently influence drug release owing to the diffusion barrier presented by the polymer. Multiple layers have been shown to provide a more stable, constant, and longer duration in comparison to blending or single-layer structures. This is observed with polymers such as ethylcellulose [19], PLLA [104], and PLGA [105], where drug release depends on the dissolution and diffusion of the polymer. 

**Figure 4:**
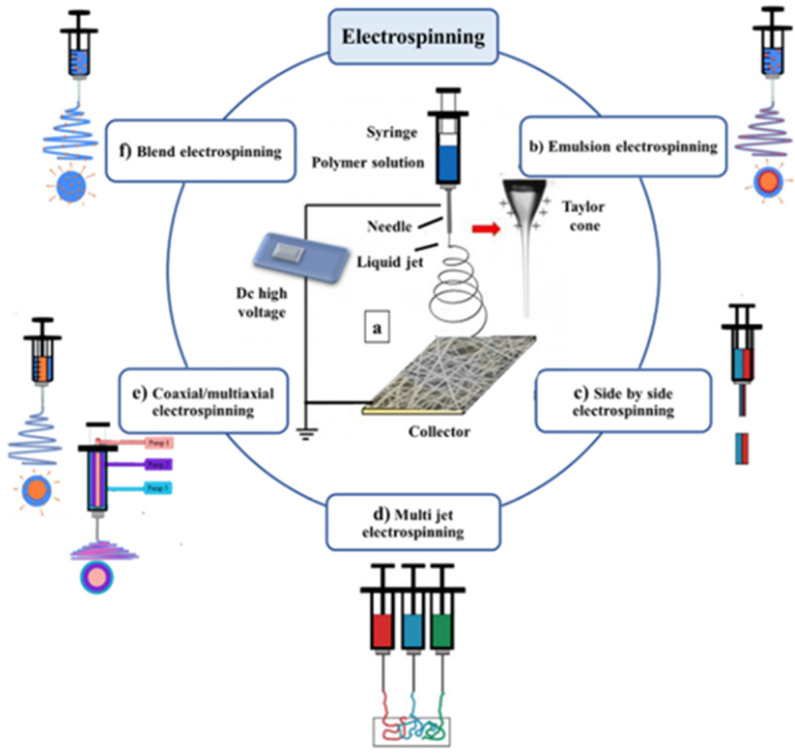
Schematic representation of the different routes to drug incorporation into a polymer carrier through electrospinning (Reproduced with permission [106]).

When the drug is sandwiched between polymer layers or present at the core of the fiber, prolonged and constant release is achievable. To achieve modification in drug release, various electrospinning techniques are applied, as shown in Figure 4. 

In blend electrospinning, both drug and polymer are dissolved into a suitable solvent, and the homogenous solution is spun into a fiber. The drug is evenly distributed in the fiber, and as a result, rapid drug release is achievable, characterized by a burst release of a drug present at the surface and which decreases gradually, approximating first-order kinetics [107].

The use of emulsion for spinning provides a means of hydrophobic polymer to carry water-soluble drugs, making it possible to encapsulate growth factors, proteins, and drugs in a polymer core [106]. The multi-jet electrospinning is ideal for large-scale production due to increased throughput. The method provides for the preparation of multicomponent fiber mats containing either multiple drugs or different fiber types. The side-by-side and coaxial/multiaxial electrospinning are also applicable methodologies depending on the desired characteristics. Electrospinning presents an opportunity to fabricate a fiber with the drug at the core and possibly achieve modified drug release. Side-by-side electrospinning presents an opportunity to combine different polymers and achieve enhanced mechanical or drug release properties [106]. 

## 5. Drug-Eluting Fibers

Drug-loaded scaffolds and sutures that deliver therapeutic agents at the wound site minimize challenges associated with systemic drug delivery, such as patient adherence, difficulty in administration, and exposure to side effects [21]. There are currently few commercially available wound dressings [49]. Common drug-eluting wound dressing materials that are commercially available include Acticoat™ (Smith & Nephew, Melbourne, Australia) and PolyMem Silver™ (Ferris, Fort Worth, Texas, USA) [108]. The former is an antimicrobial barrier silver dressing made of high-density polyethylene and nanocrystalline silver particles, while the latter is an alginate-based microbial dressing that also contains silver particles [109]. Nanofibers fabricated for wound dressings are electrospun into mesh, sheets, or mats [110]. Difficulties faced particularly with the mesh are insufficiencies in cell seeding, cell infiltration, porosity, loading efficiency, and structural stability [12]. Nanofiber mesh for wound dressing is collected on mats as unwoven matrices in an aligned or random fashion [102]. The stability and mechanical characteristics of nanofiber wound dressings are boosted with crosslinking reagents [82]. 

The first commercial drug-eluting suture is called Vicryl Plus^®^ (Ethicon, Cincinnati, Ohio, USA) and was launched in 2020; the suture is composed of a polyglactic copolymer coated with the antimicrobial triclosan [111]. A decrease in fiber diameter tends to translates into a decrease in breaking strength, ultimately negating the benefits of nanofibers. Tensile strength for sutures is paramount in tissue approximation and successful wound healing [111]. In suture fabrication, the methods of drug loading include initially electrospinning polymer material, then subsequently the suture is coated with drug; alternatively,, the sutures are spun from drug/polymer mixed solution or drug/polymer suspension. The resulting suture is either packaged as a monofilament or braided into multifilament fibers [112].

Research in fabricating drug-eluting nanofibers has gained ground, and commonly incorporated moieties include antibacterial agents, anti-inflammatories, bioadhesives, growth factors, and proliferation enhancers [49]. Table 2 provides a summary of some drug-eluting fibers that have been fabricated. 

### 5.1. Antibacterial Agents

Infection is the most common complication in wound healing; therefore, the use of antibacterial agents is essential in accelerating wound healing [36]. A study reported on the efficacy of silver-coated surgical sutures on bacterial contamination, cellular response, and wound healing using absorbable multifilament polyglactin 910 PGLA sutures with silver particles, using the in-situ synthesis and deposition technique. The study reported that the treated sutures demonstrated acceptable activity against gram-positive and negative bacteria during the degradation process, which confirmed the long-term antibacterial efficacy of the silver. The results of the scratch assay showed that the silver promoted cell migration and proliferation in the wound. The release of the silver particles increased with suture degradation [113].

A double-blind, randomised prospective pilot study on triclosan-coated sutures reported that the coating seemed to have adverse effects on wound healing. Despite handling and tension force of the sutures being comparable to standard suture material, dehiscence had a significantly higher incidence in the triclosan group than in the standard group, possibly a consequence of the formation of toxic by-products of triclosan [114]. In another study, the activity of chlorohexidine- and octenidine-coated sutures against *S. aureus*, methicillin-resistant *S. aureus* strain short MRSA, *S. epidermidis*, *Enterococcus faecalis*, and *E. coli* was assessed. The antibiotics were coated using fatty acid drug carriers onto absorbable polyglycolic acid (PGA) sutures by deep coating. The study reports that the novel coated sutures were effective against multiple species within 48 h and that the activity against *S. aureus* was superior to a commercially available antimicrobial-coated suture [115]. 

Another drug-eluting nanofiber comprising braided PLLA incorporated with cefotaxime sodium and coated with chitosan was designed. The study compared two drug loading methods and reported that the sutures showed favorable antibacterial performance; however, core–sheath sutures exhibited a relatively constant rate of drug release over a much longer duration, while the blend sutures had a burst release [104]. The rate of drug release from polymer microspheres can be tailored by controlling the thickness of the outer layer in addition to the number of layers [122]. In vivo biocompatibility tests of the cefotaxime-loaded sutures showed mild tissue reactivity in comparison to the reference [104].

A study using commercially available sutures as delivery agents for the antibacterial chitosan observed that increasing concentrations of chitosan on braided silk sutures resulted in increased knot strength; additionally, higher chitosan concentration resulted in inhibition of both *E. coli* and *S. aureus* [116].

The fabrication of sutures eluting plant extracts with antibacterial properties has also been considered by some researchers. One such study involved the incorporation of curcumin into a PLLA-based yarn suture. The study reported that the curcumin-loaded sutures exhibited superior mechanical strength, and the optimized suture released the drug in a controlled manner. Improved antibacterial properties, marked antiplatelet performance, and good biocompatibility were observed [100].

### 5.2. Anti-Inflammatory Agents

Post-operative pain originating from the wound is an outcome of inflammation associated with wound healing [105]. The presence of foreign material such as nanofibers provokes an inflammatory response [123]; hence, controlled release of an anti-inflammatory agent at the local site of surgery becomes a means of easing patient discomfort, maximizing bioavailability, and minimizing systemic exposure of the drug [105]. Nonsteroidal anti-inflammatory drugs are thus commonly used in managing post-operative pain [76].

One study considered the effect of a drug loading method on knot strength and drug release in multi- and monofilament sutures loaded with ibuprofen. The study reported that the incorporation of ibuprofen did not influence the knot assay parameters; thus, mechanical properties of the suture were maintained following drug loading. Regarding drug release, release profiles presented an initial burst with subsequent sustained release. Loading conditions like exposure time and ibuprofen concentration in coating solution did, however, influence the release rate [117].

Another study aimed at increasing the amount of ibuprofen in a suture using a novel approach of manually braiding a PLGA sheet loaded with ibuprofen around a commercialized suture. This approach provided a means of increasing drug load and tailoring drug release while maintaining the mechanical properties of the original suture [105].

Diclofenac was investigated in a similar model, in which strands cut from PLGA sheets loaded with diclofenac were braided with a commercial suture. In this study, in vitro drug release, in vivo pain relief, and histopathology were assessed. Following these characterizations, no significant differences in mechanical strength were observed between the original suture and the braided one, and drug release was reported to be sustained in the first three days for 90% of the drug while the rest was slowly released up to day 10. The in vivo pain relief evaluation results suggested that PLGA diclofenac sutures mitigated pain throughout the wound healing period via sustained release, and results of histological analysis implied that inflammatory cell recruitment and activation were suppressed [118].

### 5.3. Proliferation Enhancers

Deformities in wound contraction and compromises in collagen structure may be a consequence of inhibited cellular function [15]. Chemical mediators that enhance proliferation and mediate cellular function are required in wound healing. Examples of these substances that have shown positive in vivo and ex vivo results include MSC and transforming growth factor [32]. Studies on the delivery of growth factors by coating a commercialized suture with a drug-polymer mix have shown disparities in the results where (VEGF)-poly (D,L–lactide) (PDLLA) blend coat did not improve the meniscal healing or increase angiogenesis [119], while a VEGF/poly(L-lactide) (PLLA)-blend coating showed increased biological activity and cellular viability [120]. Differences in experimental conditions could account for the varied observations. In the former, VEGF-coated sutures were locally applied to the avascular region of the menisci in female sheep, providing an average concentration of 0.0208 µg VEGF [119]. In the latter, a running suture was inserted in the gastrocnemius muscle of male Wistar rats with concentrations of either 0.1 or 1 µg [120]. Both studies attribute dose and drug release to therapeutic outcomes. It is hypothesized that the immune response, upregulation of MMP, and downregulation of tissue inhibitors of metalloproteinases (TIMPs) induced by VEGF in the avascular menisci produced an anti-angiogenic effect [119]. These findings further justify the use of multiple therapeutic agents in wound healing.

### 5.4. Factors Impeding Industrialization of Drug-Eluting Fibers

The studies highlighted report promising results in relation to therapeutic activity in experimental models, which mostly include preclinical animal trials and in-vitro assessments. The commonly observed fabrication method of the drug-eluting fibers is a modification of commercially available fibers. Important steps in the product development of pharmaceuticals are scale-up and clinical trials. For any developed product to be of benefit to patients, the practicality of industrial development and clinical safety and efficacy should be assured. Notwithstanding, the preclinical tests and product development are critical prior steps; follow-up developmental steps are required for continued improvement in clinical practice to be actualized. Another important consideration in the production process would be the influence of the sterilization process on the integrity of the polymer systems [21]. Primary wound dressings and sutures are required to be sterile; therefore, the effect of sterilization processes on the stability and performance of drug-eluting fibers should be considered. The release profile of the therapeutic agent is also an important factor to consider seeing that an initial burst release of antimicrobial agent may be required to prevent microbial infection [123], while the sustained release of anti-inflammatories may be ideal for alleviating pain during the healing period [118]. Consequently, the choice of drug carrier becomes crucial depending on the desired release profile.

Another encountered challenge in designing drug-eluting fibers is limited drug loading, particularly observed for cells and large molecules [70]. A limited drug load restricts drug choice only to potent products to achieve appreciable pharmacological effects [21]. To counter the negative effect of drug incorporation into fibers, some researchers consider coating or braiding commercially available sutures already possessing ideal mechanical properties with drug-loaded polymer [117]. The limitation of modifying a commercially available suture into a drug-eluting device is the practicality of the application of the process in clinical practice. The approach provides a means of increasing drug load and tailoring drug release while maintaining the mechanical properties of the original suture. In addition, the process is not scalable, thereby limiting applicability [105]. An ideal situation would be the incorporation of the coating step at the production level, which would provide an analysis of potential influences on process parameters, the practicality of scale-up, and the stability of the final product. 

Alternatively, practical and reproducible methods of fiber modification tailored for health institutions can be developed. Despite the soaking method being simple, the need to dry the solvent increases the number of unit steps adding to the cost of production and risk of compromised stability. Solvent choice also needs to be accurate since it should be able to swell the polymer matrix to enhance impregnation of the drug without dissolving the suture also, the residue solvent amounts should be within acceptable limits [20].

## 6. Economic Considerations

Non-healing wounds present a huge economic challenge and are estimated to affect 120 per 100,000 people between 45 and 65 years, rising to 800 per 100,000 people for those above 75 years [13]. Non-healing surgical wounds represent the largest category averaging approximately 20% in the United States [124] and 35% in Canada [125]. Improvement in wound care management and strategies to minimize infection is paramount if health costs relating to wound management are to be reduced. A prospective study that evaluated the impact of restructuring wound management practices submitted that utilization of advanced dressings based on moist wound healing principles resulted in >70% reduction in total cost per patient for 50 matched patient samples in 2005 and 2006. Advanced healing practices were also reported to shorten wound healing time by 33 weeks (60%) [107]. The cost of healing is said to increase with the duration of healing. The ideal approach to inducing and accelerating wound healing is a combination of cell therapy, therapeutic agents, and scaffolding material [68]. 

Ideally, this combined approach would facilitate timely healing and prevent progression to chronic wounds that present an economic burden. The practicality of cell therapy is currently limited; however, advances have been made in fabricating drug-eluting scaffolds, with a few of these products becoming commercially available. The emergence of nanofibers by electrospinning presents an opportunity to improve wound management and reduce the cost associated with chronic wound care as a result of the inherent advantages of nanofibers relating to porosity, reduced diameter, and similarity to ECM morphology that are vital for wound healing. Further, the fabrication process is simple, cost-effective, and versatile, allowing for the incorporation of therapeutic agents, optimization, and scale-up with possible industrialization and clinical application [126].

Recently developed wound dressings and sutures were designed to influence the microenvironment of the wound site to stimulate healing and/or prevent complications as opposed to merely covering or approximating tissues as with traditional dressings [14]. Despite the cost of modern dressings being relatively higher than traditional dressings, modern dressings have better efficacy in terms of wound healing, potentially resulting in a lower total cost of wound healing. Among the contributing factors to cost reductions are the reported decreases in changing frequency and pain scale, as well as increased patient comfort [108].

## 7. Future Perspectives and Conclusions

Minimizing complications in the healing process of surgical wounds requires optimization of structural and pharmacological adjuvants to accelerate wound healing. On-site drug delivery using systems with ideal mechanical properties present an efficient means of accelerating wound healing. Due to the complexity of wound healing, classes of pharmacological agents required for successful wound healing include, but are not limited to, antimicrobial, anti-inflammatory, and proliferation enhancing agents. Current processing methods of loading scaffolding and suture material with the drug dispersed in polymer carriers provide an avenue for site-specific drug delivery with the possibility of modifying the release rate. However, difficulties in regulating polymer degradation rate, loading and distribution of therapeutic agents, biocompatibility, and obtaining clinical data, to name a few, impede further exploration and maximization of these delivery systems. 

The utilization of the electrospinning technique to develop multicomponent drug-eluting nanofibers, upscaling already fabricated nanofibers with promising results, and progressing to clinical trials are avenues worth pursuing to improve the odds of successful healing of surgical wounds and ultimately enhancing the quality of patient care. The consideration of possible limitations, such as fiber size limiting the maximum drug load, compromise of mechanical properties, and an alteration of the integrity of polymer-building blocks by a sterilization process, is required during product development to increase the chances of clinical applicability and industrialization.

## Figures and Tables

**Table 1:** Reported incidence of SSI up to 30 days post-surgery.

Country	Overall Infection Rate *	SSI Detection (%)				Ref
		a	b	c	x	
United States	6.8	51.9		44.4	3.7	[57]
United States	16	78	9	13		[58]
Hungary	2.9	24	6	70		[60]
Kosovo	12	40.7	3.7	55.6		[61]
Algeria	5.4	62		38		[62]
Canada	3.9	15.8	29.2	57.5		[63]
United Kingdom	15.6	14		86		[64]
Kosovo	9.8	6.3		93.7		[65]
Saudi Arabia	16.3	9		45	45	[66]

* Per 100 procedures, ^a^ deep incisional, ^b^ organ-specific, ^c^ superficial/skin, ^x^ other.

**Table 2:** Summary of studies relating to fabricated drug-eluting fibers.

Therapeutic Agent/Class	Drug Carrier	Treatment Method	Therapeutic Activity Assessment Method	General Findings
Silver particles/antibacterial	Silver solution on 910 PGLA suture	Silver deposition technology	In vitro	Modified sutures demonstrated long-term antibacterial capability on Gram-positive and Gram-negative bacteria [113]
Triclosan/antibacterial	Vicryl Plus ^®^ (Ethicon, Cincinnati, Ohio, USA)	Coating	Double-blind randomized prospective pilot study	Toxic byproducts of triclosan possibly adversely affected wound healing [114]
Chlorohexidine/Octenidine (antibacterial)	PA80/LA80 on Gunze PGA suture (Gunze Limited, Tokyo, Japan)	Dip coating	In vitro	Coated sutures were effective against multiple species within 48 h [115]
Cefotaxime & Chitosan (antibacterial)	PLLA	Electrospinning (cefotaxime: core-sheath or blend), braiding and dipping in chitosan solution	In vitro	Constant drug release was observed for core-sheath. Mild tissue reactivity [104]
Chitosan (antibacterial)	Braided silk sutures	Coating	In vitro	Increased knot strength of suture, both *E. coli* and *S aureus* were inhibited [116]
Curcumin hydrochloride (antibacterial/anti-inflammatory)	PLLA	Electrospinning (curcumin hydrochloride blend)	Preclinical	Curcumin-loaded sutures exhibited superior mechanical strength. Optimized suture released the drug in a controlled manner. Improved antibacterial properties, marked antiplatelet performance, and good biocompatibility were observed [100]
Ibuprofen (anti-inflammatory)	Braided polyglycolide thread/Poly(p-dioxanone) monofilaments	Coating	N/A	Drug release began with initial burst followed by then sustained release [117]
Ibuprofen (anti-inflammatory)	PLGA sheets braided on VICRYL™W9114 suture (Ethicon, Cincinnati, Ohio, USA )	Electrospinning (single/multiple layered sheets)	Preclinical	Drug loading was reproducible, multiple layers prolonged drug release. Pain relief efficacy similar to oral drug administration. Fabrication method is not scalable [105]
Diclofenac (anti-inflammatory)	PLGA sheets braided on 3-0 VICRYL™W9114 suture (Ethicon, Cincinnati, Ohio, USA )	Electrospinning	Preclinical	Sustained drug release was attained. Pain was mitigated throughout the wound healing period. Recruitment of inflammatory cells was suppressed [118].
VEGF (Proliferation enhancer)	PDLLA (VEGF blend) on Ethibond™ suture (Ethicon, Cincinnati, Ohio, USA )	Coating	Preclinical	Meniscal healing did not improve, and angiogenesis did not increase [119]
VEGF (Proliferation enhancer)	PLLA (VEGF blend) on EthiconPDS™ suture (Ethicon, Cincinnati, Ohio, USA )	Coating	Preclinical	Biological activity and cellular viability increased [120]
Norepinephrine/dopamine (bioadhesive)	Collagen-CaCO_3_ PNE composite scaffold	Electrospinning & complexation	In vitro	Satisfactory cellular adhesion, proliferation and differentiation of human fetal osteoblasts. Potential osteoconductive scaffolds for bone tissue engineering [121]

## Data Availability

The data presented in this study are available on request from the corresponding author.

## References

[1] Liu X., Xu H., Zhang M., Yu D.G. (2021). Electrospun medicated nanofibers for wound healing: Review. Membranes.

[2] Gilaberte Y., Prieto-Torres L., Pastushenko I., Juarranz Á. (2016). Anatomy and Function of the Skin.

[3] Monteiro-Riviere N.A. (2010). Structure and function of skin. Toxicology of the Skin.

[4] Yang J., Wang K., Yu D.G., Yang Y., Bligh S.W.A., Williams G.R. (2020). Electrospun Janus nanofibers loaded with a drug and inorganic nanoparticles as an effective antibacterial wound dressing. Mater. Sci. Eng. C.

[5] Najafi S., Gholipour-Kanani A., Eslahi N., Bahrami S.H. (2021). Study on release of cardamom extract as an antibacterial agent from electrospun scaffold based on sodium alginate. J. Text. Inst..

[6] Schiff B.A. (2009). Wound Healing. Complications in Head and Neck Surgery.

[7] Peng Y., Ma Y., Bao Y., Liu Z., Chen L., Dai F., Li Z. (2021). Electrospun PLGA/SF/artemisinin composite nanofibrous membranes for wound dressing. Int. J. Biol. Macromol..

[8] Lorenz H.P., Longaker M.T. (2003). Wounds: Biology, Pathology, and Management. Essential Practice of Surgery.

[9] Nussbaum S.R., Carter M.J., Fife C.E., DaVanzo J., Haught R., Nusgart M., Cartwright D. (2018). An Economic Evaluation of the Impact, Cost, and Medicare Policy Implications of Chronic Nonhealing Wounds. Value Health.

[10] Debas H.T., Gosselin R., Mccord C. (2004). Surgery. Disease Control Priorities in Developing Countries.

[11] Yao K., Bae L., Yew W.P. (2013). Post-operative wound management. Aust. Fam. Physician.

[12] Singhal H., Kaur K. Wound Infection. Medscape: General Surgery. Medscape, Manhattan, 2021. https://emedicine.medscape.com/article/188988-overview#a4.

[13] Velnar T., Bailey T., Smrkolj V. (2009). The wound healing process: An overview of the cellular and molecular mechanisms. J. Int. Med. Res..

[14] Dhivya S., Padma V.V., Santhini E. (2015). Wound dressings—A review. BioMedicine.

[15] Chaudhary C., Garg T. (2015). Scaffolds: A novel carrier and potential wound healer. Crit. Rev. Ther. Drug Carr. Syst..

[16] Wei Z., Wang L., Zhang S., Chen T., Yang J., Long S., Wang X. (2020). Electrospun antibacterial nanofibers for wound dressings and tissue medicinal fields: A Review. J. Innov. Opt. Health Sci..

[17] El Fawal G., Hong H., Mo X., Wang H. (2021). Fabrication of scaffold based on gelatin and polycaprolactone (PCL) for wound dressing application. J. Drug Deliv. Sci. Technol..

[18] Ramazan E. (2019). Advances in Fabric Structures for Wound Care.

[19] Senthamizhan A., Balusamy B., Uyar T. (2017). Electrospinning: A Versatile Processing Technology for Producing Nanofibrous Materials for Biomedical and Tissue-Engineering Applications.

[20] Arora A., Aggarwal G., Chander J., Maman P., Nagpal M. (2019). Drug eluting sutures: A recent update. J. Appl. Pharm. Sci..

[21] Luk A. (2013). Critical challenges to the design of drug-eluting medical devices. Ther. Deliv..

[22] Ruiz M.E., Montoto S.S., Talevi A., Quiroga P.A.M. (2018). Routes of Drug Administration. ADME Processes in Pharmaceutical Sciences.

[23] Parikh K.S. (2017). Nano-Structured, Drug Eluting Medical Devices For Improved Clinical Outcomes. Ph.D. Thesis.

[24] Anselmo A.C., Mitragotri S. (2014). An overview of clinical and commercial impact of drug delivery systems. J. Control. Release.

[25] Guo S., DiPietro L.A. (2010). Critical review in oral biology & medicine: Factors affecting wound healing. J. Dent. Res..

[26] Schultz G.S., Chin G.A., Moldawer L., Diegelmann R.F. (2011). Principles of wound healing. Mechanisms of Vascular Disease: A Reference Book for Vascular Specialists.

[27] Rang H.P., Dale M.M., Ritter J.M., Flower R.J., Henderson G. (2012). Rang and Dale’s Pharmacology.

[28] (2003). Maureane Hoffman Remodeling the Blood Coagulation Cascade. J. Thromb. Thrombolysis.

[29] Higgins R.A. (2016). Coagulation Pathway and Physiology. Hemostasis Physiology.

[30] Chaudhry R., Usama S.M., Babiker H.M. (2021). Physiology, Coagulation Pathways.

[31] Perez-Pujol S., Aras O., Escolar G. (2012). Factor V Leiden and Inflammation. Thrombosis.

[32] Marshall C.D., Hu M.S., Leavitt T., Barnes L.A., Lorenz H.P., Longaker M.T. (2018). Cutaneous Scarring: Basic Science, Current Treatments, and Future Directions. Adv. Wound Care.

[33] Broughton G., Janis J.E., Attinger C.E. (2006). Wound healing: An overview. Plast. Reconstr. Surg..

[34] ben Amar M., Wu M. (2014). Re-epithelialization: Advancing epithelium frontier during wound healing. J. R. Soc. Interface.

[35] Gonzalez A.C.D.O., Andrade Z.D.A., Costa T.F., Medrado A.R.A.P. (2016). Wound healing—A literature review. An. Bras. Dermatol..

[36] Wolcott R., Cutting K., Dowd S., Percival S. (2010). Types of Wounds and Infections. Microbiology Wounds.

[37] Emanuele A.S., Giada M., Alessio F., Ciprandi G. (2019). From Tissue Repair To Tisse Regenetaion. Wound Healing.

[38] Lipsky B.A., Hoey C. (2009). Topical antimicrobial therapy for treating chronic wounds. Clin. Infect. Dis..

[39] Rathur H.M., Boulton A.J.M. (2005). Recent advances in the diagnosis and management of diabetic neuropathy. J. Bone Jt. Sur. Ser. B.

[40] Webb R. (2017). Pressure ulcer over pressure injury. Br. J. Nurs..

[41] Stavrou D., Weissman O., Tessone A., Zilinsky I., Holloway S., Boyd J., Haik J. (2014). Health Related Quality of Life in burn patients—A review of the literature. Burns.

[42] Gianfaldoni S., Wollina U., Lotti J., Gianfaldoni R., Lotti T., Fioranelli M., Roccia M.G. (2017). History of venous leg ulcers. J. Biol. Regul. Homeost. Agents.

[43] Kirkwood M.L., Arbique G.M., Guild J.B., Timaran C., Chung J., Modrall G., Anderson J.A., Valentine R.J. (2013). Radiation Skin Injury: More Frequent After Complex Endovascular Procedures?. J. Vasc. Surg..

[44] Coruh A., Yontar Y. (2012). Application of split-thickness dermal grafts in deep partial- and full-thickness burns: A new source of auto-skin grafting. J. Burn Care Res..

[45] Amala C. (2020). Necrosis and Types of Necrosis. Trans. Med..

[46] Williams J.Z., Barbul A. (2003). Nutrition and wound healing. Surg. Clin. N. Am..

[47] Barchitta M., Maugeri A., Favara G., San Lio R.M., Evola G., Agodi A., Basile G. (2019). Nutrition and wound healing: An overview focusing on the beneficial effects of curcumin. Int. J. Mol. Sci..

[48] Nuutila K., Eriksson E. (2021). Moist Wound Healing with Commonly Available Dressings. Adv. Wound Care.

[49] Théorêt C., Stashak T. (2011). Wound dressing. Clinical Veterinary Advisor.

[50] Bayat A., McGrouther D.A., Ferguson M.W.J. (2003). Skin scarring. Br. Med. J..

[51] Larson B.J., Longaker M.T., Lorenz P.H. (2010). Scarless Fetal Wound Healing: A Basic Science Review. Plast. Reconstr. Surg..

[52] Colwell A.S., Longaker M.T., Lorenze P.H. (2003). Fetal Wound Healing. Front. Biosci..

[53] Stöppler M.C. Medterms Medical Dictionary a-z list/ Surgery Definition. https://www.medicinenet.com/surgery/definition.htm.

[54] Bae J.Y., Groen R.S., Kushner A.L. (2011). Surgery as a public health intervention: Common misconceptions versus the truth. Bull. World Health Organ..

[55] Liu Z., Dumville J.C., Norman G., Westby M.J., Blazeby J., McFarlane E., Welton N.J., O’Connor L., Cawthorne J., George R.P. (2018). Intraoperative interventions for preventing surgical site infection: An overview of Cochrane Reviews. Cochrane Database Syst. Rev..

[56] Mangram A.J., Horan T.C., Pearson M.L., Christine Silver L., Jarvis W.R., The Hospital Infection Control Practices Advisory Committee (1999). Guideline for Prevention of Surgical Site Infection, 1999. Infect. Control Hosp. Epidemiol..

[57] Onyekwelu I., Yakkanti R., Protzer L., Pinkston C.M., Tucker C., Seligson D. (2017). Surgical Wound Classification and Surgical Site Infections in the Orthopaedic Patient. J. Am. Acad. Orthop. Surg. Glob. Res. Rev..

[58] Gaynes R.P., Culver D.H., Horan T.C., Edwards J.R., Richards C., Tolson J.S. (2001). Surgical Site Infection (SSI) rates in the United States, 1992-1998: The National Nosocomial Infections Surveillance system basic SSI risk index. Clin. Infect. Dis..

[59] Goyal R., Sandhu H.P.S., Kumar A., Kosey S. (2015). Surgical Site Infection in General Surgery. Int. J. Sci. Res. Knowl..

[60] Gulacsi L., Tatar Kiss Z., Goldmann D.A., Huskins W.C. (2000). Risk-adjusted infection rates in surgery: A model for outcome measurement in hospitals developing new quality improvement programmes. J. Hosp. Infect..

[61] Raka L., Krasniqi A., Hoxha F., Musa R., Mulliqi G., Krasniqi S., Kurti A., Dervishaj A., Nuhiu B., Kelmendi B. (2007). Surgical site infections in an abdominal surgical ward at Kosovo Teaching Hospital. J. Infect. Dev. Ctries..

[62] Atif M.L., Azouaou A., Bouadda N., Bezzaoucha A., Si-Ahmed M., Bellouni R. (2015). Incidence and predictors of surgical site infection in a general surgery department in Algeria. Rev. d’Epidemiol. Sante Publique.

[63] van Walraven C., Musselman R. (2013). The Surgical Site Infection Risk Score (SSIRS): A Model to Predict the Risk of Surgical Site Infections. PLoS ONE.

[64] Woldemicael A.Y., Bradley S., Pardy C., Richards J., Trerotoli P., Giuliani S. (2019). Surgical Site Infection in a Tertiary Neonatal Surgery Centre. Eur. J. Pediatric Surg..

[65] Zejnullahu V.A., Zejnullahu V.A., Isjanovska R., Sejfija Z. (2019). Surgical site infections after cesarean sections at the University Clinical Center of Kosovo: Rates, microbiological profile and risk factors. BMC Infect. Dis..

[66] Alkaaki A., Al-Radi O.O., Khoja A., Alnawawi A., Alnawawi A., Maghrabi A., Altaf A., Aljiffry M. (2019). Surgical site infection following abdominal surgery: A prospective cohort study. Can. J. Surg..

[67] Shi C., Wang C., Liu H., Li Q., Li R., Zhang Y., Liu Y., Shao Y., Wang J. (2020). Selection of Appropriate Wound Dressing for Various Wounds. Front. Bioeng. Biotechnol..

[68] Nour S., Baheiraei N., Imani R., Khodaei M., Alizadeh A., Rabiee N., Moazzeni S.M. (2019). A review of accelerated wound healing approaches: Biomaterial- assisted tissue remodeling. J. Mater. Sci. Mater Med..

[69] Aronson A., Laageide L., Powers J. (2018). Use of Stem Cells in Wound Healing. Curr. Dermatol. Rep..

[70] Lee D.E., Ayoub N., Agrawal D.K. (2016). Mesenchymal stem cells and cutaneous wound healing: Novel methods to increase cell delivery and therapeutic efficacy. Stem Cell Res. Ther..

[71] Nourian Dehkordi A., Mirahmadi Babaheydari F., Chehelgerdi M., Raeisi Dehkordi S. (2019). Skin tissue engineering: Wound healing based on stem-cell-based therapeutic strategies. Stem Cell Res. Ther..

[72] Das S., Baker A.B. (2016). Biomaterials and nanotherapeutics for enhancing skin wound healing. Front. Bioeng. Biotechnol..

[73] Grazul-Bilska A.T., Johnson M.L., Bilski J.J., Redmer D.A., Reynolds L.P., Abdullah A., Abdullah K.M. (2003). Wound healing: The role of growth factors. Drugs Today.

[74] Eming S.A., Krieg T., Davidson J.M. (2007). Gene Therapy and Wound Healing. Clin. Dermatol..

[75] Punjataewakupt A., Napavichayanun S., Aramwit P. (2019). The downside of antimicrobial agents for wound healing. Eur. J. Clin. Microbiol. Infect. Dis..

[76] Krischak G.D., Augat P., Claes L., Kinzl L., Beck A. (2007). The effects of non-steroidal anti-infl ammatory drug application on incisional wound healing in rats. J. Wound Care.

[77] Su W.H., Cheng M.H., Lee W.L., Tsou T.S., Chang W.H., Chen C.-S., Wang P.H. (2010). Nonsteroidal anti-inflammatory drugs for wounds: Pain relief or excessive scar formation?. Mediat. Inflamm..

[78] Tarafder S., Park G.Y., Felix J., Lee C.H. (2020). Bioadhesives for musculoskeletal tissue regeneration. Acta Biomater..

[79] Duan W., Bian X., Bu Y. (2021). Applications of Bioadhesives: A Mini Review. Front. Bioeng. Biotechnol..

[80] Ge L., Chen S. (2020). Recent advances in tissue adhesives for clinical medicine. Polymers.

[81] Duarte A.P., Coelho J.F., Bordado J.C., Cidade M.T., Gil M.H. (2012). Surgical adhesives: Systematic review of the main types and development forecast. Prog. Polym. Sci..

[82] Boateng J.S., Matthews K.H., Stevens H.N.E., Eccleston G.M. (2008). Wound healing dressings and drug delivery systems: A review. J. Pharm. Sci..

[83] Agarwal Y., Rajinikanth P.S., Ranjan S., Tiwari U., Balasubramnaiam J., Pandey P., Arya D.K., Anand S., Deepak P. (2021). Curcumin loaded polycaprolactone-/polyvinyl alcohol-silk fibroin based electrospun nanofibrous mat for rapid healing of diabetic wound: An in-vitro and in-vivo studies. Int. J. Biol. Macromol..

[84] Nagar H.K., Srivastava A.K., Srivastava R., Kurmi M.L., Chandel H.S., Ranawat M.S. (2016). Pharmacological Investigation of the Wound Healing Activity of Cestrum nocturnum (L.) Ointment in Wistar Albino Rats. J. Pharm..

[85] Su C., Menon N.V., Xu X., Teo Y.R., Cao H., Dalan R., Tay C.Y., Hou H.W. (2021). A novel human arterial wall-on-a-chip to study endothelial inflammation and vascular smooth muscle cell migration in early atherosclerosis. Lab Chip.

[86] Nugud A., Sandeep D., El-Serafi A.T. (2018). Two faces of the coin: Minireview for dissecting the role of reactive oxygen species in stem cell potency and lineage commitment. J. Adv. Res..

[87] Cao H., Yang L., Tian R., Wu H., Gu Z., Li Y. (2022). Versatile polyphenolic platforms in regulating cell biology. Chem. Soc. Rev..

[88] Zhang X., Li Z., Yang P., Duan G., Liu X., Gu Z., Li Y. (2021). Polyphenol scaffolds in tissue engineering. Mater. Horiz..

[89] Hu J., Yang L., Yang P., Jiang S., Liu X., Li Y. (2020). Polydopamine free radical scavengers. Biomater. Sci..

[90] Ou Q., Zhang S., Fu C., Yu L., Xin P., Gu Z., Cao Z., Wu J., Wang Y. (2021). More natural more better: Triple natural anti-oxidant puerarin/ferulic acid/polydopamine incorporated hydrogel for wound healing. J. Nanobiotechnol..

[91] Gathani K.M., Raghavendra S.S. (2016). Scaffolds in regenerative endodontics: A review. Dent. Res. J..

[92] Samadian H., Zamiri S., Ehterami A., Farzamfar S., Vaez A., Khastar H., Alam M., Ai A., Derakhshankhah H., Allahyari Z. (2020). Electrospun cellulose acetate/gelatin nanofibrous wound dressing containing berberine for diabetic foot ulcer healing: In vitro and in vivo studies. Sci. Rep..

[93] Sabbagh F., Kim B.S. (2022). Recent advances in polymeric transdermal drug delivery systems. J. Control. Release.

[94] Vyas S., Zhang X., Goli E., Geubelle P.H. (2020). Frontal vs. bulk polymerization of fiber-reinforced polymer-matrix composites. Compos. Sci. Technol..

[95] Ghaemi N., Safari P. (2018). Nano-porous SAPO-34 enhanced thin-film nanocomposite polymeric membrane: Simultaneously high water permeation and complete removal of cationic/anionic dyes from water. J. Hazard. Mater..

[96] Çaykara T., Akçakaya İ. (2006). Synthesis and network structure of ionic poly(N,N-dimethylacrylamide-co-acrylamide) hydrogels: Comparison of swelling degree with theory. Eur. Polym. J..

[97] Buyukgoz G.G., Castro J.N., Atalla A.E., Pentangelo J.G., Tripathi S., Davé R.N. (2021). Impact of Mixing on Content Uniformity of Thin Polymer Films Containing Drug Micro-Doses. Pharmaceutics.

[98] Guillen G.R., Pan Y., Li M., Hoek E.M. (2011). Preparation and Characterization of Membranes Formed by Nonsolvent Induced Phase Separation: A Review. Ind. Eng. Chem. Res..

[99] Lee W.R., Im C., Park H.-Y., Seo J.-M., Kim J.-M. (2019). Fabrication of Convex PDMS–Parylene Microstructures for Conformal Contact of Planar Micro-Electrode Array. Polymers.

[100] Richard A.S., Verma R.S. (2021). Bioactive nano yarns as surgical sutures for wound healing. Mater. Sci. Eng. C.

[101] Boybeyi Ö., Kaçmaz B., Günal Y.D., Gül S., Yörübulut S., Aslan M.K. (2016). Bacterial adhesion to braided surgical sutures: An in vitro study. Eur. J. Plast. Surg..

[102] Keshvardoostchokami M., Majidi S.S., Huo P., Ramachandran R., Chen M., Liu B. (2021). Electrospun nanofibers of natural and synthetic polymers as artificial extracellular matrix for tissue engineering. Nanomaterials.

[103] Alghoraibi I., Alomari S. (2020). Different Methods for Nanofiber Design and Fabrication.

[104] Hu W., Huang Z.M., Liu X.Y. (2010). Development of braided drug-loaded nanofiber sutures. Nanotechnology.

[105] Lee J.E., Park S., Park M., Kim M.H., Park C.G., Lee S.H., Choi S.Y., Kim B.H., Park H.J., Park J.H. (2013). Surgical suture assembled with polymeric drug-delivery sheet for sustained, local pain relief. Acta Biomater..

[106] Zare M., Dziemidowicz K., Williams G.R., Ramakrishna S. (2021). Encapsulation of pharmaceutical and nutraceutical active ingredients using electrospinning processes. Nanomaterials.

[107] Amarjargal A., Brunelli M., Fortunato G., Spano F., Kim C.-S., Rossi R.M. (2019). On-demand drug release from tailored blended electrospun nanofibers. J. Drug Deliv. Sci. Technol..

[108] Mousavi S.M., Nejad Z.M., Hashemi S.A., Salari M., Gholami A., Ramakrishna S., Chiang W.H., Lai C.W. (2021). Bioactive agent-loaded electrospun nanofiber membranes for accelerating healing process: A review. Membranes.

[109] Boonkaew B., Kempf M., Kimble R., Supaphol P., Cuttle L. (2014). Antimicrobial efficacy of a novel silver hydrogel dressing compared to two common silver burn wound dressings: Acticoat^TM^ and PolyMem Silver®. Burns.

[110] Fahimirad S., Abtahi H., Satei P., Ghaznavi-Rad E., Moslehi M., Ganji A. (2021). Wound healing performance of PCL/chitosan based electrospun nanofiber electrosprayed with curcumin loaded chitosan nanoparticles. Carbohydr. Polym..

[111] Alshomer F., Madhavan A., Pathan O., Song W. (2017). Bioactive Sutures: A Review of Advances in Surgical Suture Functionalisation. Curr. Med. Chem..

[112] Deng X., Qasim M., Ali A. (2021). Engineering and polymeric composition of drug-eluting suture: A review. J. Biomed. Mater. Res. Part A.

[113] Gallo A.L., Paladini F., Romano A., Verri T., Quattrini A., Sannino A., Pollini M. (2016). Efficacy of silver coated surgical sutures on bacterial contamination, cellular response and wound healing. Mater. Sci. Eng. C.

[114] Deliaert A.E., van den Kerckhove E., Tuinder S., Fieuws S., Sawor J.H., Meesters-Caberg M.A., van der Hulst R.R. (2009). The effect of triclosan-coated sutures in wound healing. A double blind randomised prospective pilot study. J. Plast. Reconstr. Aesthetic Surg..

[115] Obermeier A., Schneider J., Föhr P., Wehner S., Kühn K.D., Stemberger A., Schieker M., Burgkart R. (2015). In vitro evaluation of novel antimicrobial coatings for surgical sutures using octenidine. BMC Microbiol..

[116] Viju S., Thilagavathi G. (2013). Effect of chitosan coating on the characteristics of silk-braided sutures. J. Ind. Text..

[117] Zurita R., Puiggalí J., Rodríguez-Galán A. (2006). Loading and release of ibuprofen in multi- and monofilament surgical sutures. Macromol. Biosci..

[118] Huh B.K., Kim B.H., Kim S.N., Park C.G., Lee S.H., Kim K.R., Heo C.Y., Choy Y. (2017). bin Surgical suture braided with a diclofenac-loaded strand of poly(lactic-co-glycolic acid) for local, sustained pain mitigation. Mater. Sci. Eng. C.

[119] Kopf S., Birkenfeld F., Becker R., Petersen W., Stärke C., Wruck C.J., Tohidnezhad M., Varoga D., Pufe T. (2010). Local treatment of meniscal lesions with vascular endothelial growth factor. J. Bone Jt. Surg. Ser. A.

[120] Bigalke C., Luderer F., Wulf K., Storm T., Lobler M., Arbeiter D., Rau B.M., Nizze H., Vollmar B., Schmitz K.P. (2014). VEGF-releasing suture material for enhancement of vascularization: Development, in vitro and in vivo study. Acta Biomater..

[121] Dhand C., Ong S.T., Dwivedi N., Diaz S.M., Venugopal J.R., Navaneethan B., Fazil M.H.U.T., Liu S., Seitz V., Wintermantel E. (2016). Bio-inspired in situ crosslinking and mineralization of electrospun collagen scaffolds for bone tissue engineering. Biomaterials.

[122] Uttarwar M., Aswath P. (2008). Fabrication of porous, drug-releasing, biodegradable, polymer scaffolds for sustained drug release. J. Biomed. Mater. Res. Part B Appl. Biomater..

[123] He C.L., Huang Z.M., Han X.J. (2009). Fabrication of drug-loaded electrospun aligned fibrous threads for suture applications. J. Biomed. Mater. Res. Part A.

[124] Fife C.E., Carter M.J., Walker D., Thomson B. (2012). Wound Care Outcomes and Associated Cost Among Patients Treated in US Outpatient Centers: Data from the US Wound Registry. Wound Care Learn. Netw..

[125] Hurd T., Zuiliani N., Posnett J. (2008). Evaluation of the impact of restructuring wound management practices in a community care provider in Niagara, Canada. Int. Wound J..

[126] El-aassar M.R., Ibrahim O.M., Al-oanzi Z.H. (2021). Biotechnological applications of polymeric nanofiber platforms loaded with diverse bioactive materials. Polymers.

